# Acute compartment syndrome of the hand in Henoch-Schonlein Purpura

**DOI:** 10.1186/1752-1947-1-6

**Published:** 2007-03-02

**Authors:** Guntur E Luis, Eng-Seng Ng

**Affiliations:** 1 Department of Orthopedics Surgery, Faculty of Medicine, University of Malaya, Kuala Lumpur, Malaysia

## Abstract

An eight year old boy with Henoch-Schonlein Purpura (HSP) presented with acute compartment syndrome (ACS) of his left hand following arterial cannulation of his radial artery in intensive care unit. Emergency decompression and fasciotomy were performed. The authors report this first case in literature and discuss how HSP can be complicated by ACS and ways to prevent the latter from happening.

## Background

Henoch-Schonlein Purpura is one of the most common vasculitides of childhood and is considered to be self-limiting. One manifestation of HSP that can continue to cause lifelong problems is renal involvement [1].

In 1990, the American College of Rheumatology published diagnostic criteria for HSP. These included (1) Palpable purpura-slightly raised "palpable" hemorrhagic skin lesions, not related to thrombocytopenia; (2) Age less than 20 at disease onset-patient 20 years or younger at onset of first symptoms; (3) Bowel angina-diffuse abdominal pain, worse after meals, or the diagnosis of bowel ischemia, usually including bloody diarrhea; and (4) Wall granulocytes on biopsy-histologic changes showing granulocytes in the walls of arterioles or venules. The classification further states, "For purposes of classification, a patient shall be said to have Henoch-Schonlein purpura if at least 2 of these 4 criteria are present. The presence of any 2 or more criteria yields a sensitivity of 87.1% and a specificity of 87.7%" [2].

## Case report

An eight year old Malay boy, with a history of Henoch-Schonlein Purpura and G-6-PD deficiency, presented with left hand swelling and punctate rashes on the dorsum of his left hand, four hours following his transfer out from intensive care unit (ICU).

The history dated back to two weeks prior to admission when he noted rashes on the dorsum of his feet and had intermittent, diffuse abdominal pain. He then developed migratory pain in his ankle joints, knee, elbow and small finger joints in that order. He refused treatment.

He had no drug allergies. Developmental milestones were normal and immunization was complete.

A week later, he was admitted to the general pediatrics ward for intermittent vomiting, severe generalized abdominal pain and passing red-current stools. The pain was constant, unrelenting and aggravated by solid food intake despite intravenous omeprazole (16 mg bd), metoclopromide (3 mg tds) and tramadol (30 mg 4 hourly). Rashes now developed on his left flank, buttock and medial aspects of both feet. He was febrile and micturation was normal.

His vital parameters were as follows: BP 127/96, PR 99/min, RR 24/min and SpO2 97% on room air. Clinical examination revealed non-blanching purpuric rashes on the dorsum of his feet, left flank and buttock [Fig. 3]. There was also localized tenderness on deep palpation of his left iliac fossa. Hematological, coagulation and renal profiles were within normal limits.

Urgent transabdominal ultrasound did not show any "pseudokidney" or "doughnut sign" to suggest intussusception." Peristalsis was normal. All intraabdominal organs were normal and there was no free fluid. There was, in the left iliac fossa, a bowel loop filled with echogenic material likely to be a stool."

Thirty-six hours later, he was admitted to the pediatric ICU owing to poor intake, severe per rectal bleeding and deteriorating general conditions. Fluid challenge with 300 mls of Ringer's Lactate solution and 2 units of packed red cell transfusion were given. Intra-arterial cannulation of the left radial artery was performed for continuous blood-pressure monitoring. Intravenous fluids, methylprednisolone (16 mg od), PCA morphine (bolus 0.5 mg, lockout at 15 mg), intravenous ceftriaxone (800 mg od) and metronidazole (120 mg tds) were administered.

Following two days of stabilization, he was transferred out from ICU after the removal of his arterial line and urine catheter. Clinical examination in the morning showed diffuse, oedematous swelling on the dorsum of the left hand and all fingers with non-blanching purpuric rash restricted to an area 4 cm in diameter. There were no signs of fluid extravasations, inflammation or infection at the site of cannulation.

An urgent Doppler ultrasound of the left forearm was performed to exclude a thrombo-embolic event. Both radial and ulnar arteries and corresponding veins were patent. Normal Doppler flow pattern was obtained within these vessels up to the level of cubital fossa.

**Figure 1:**
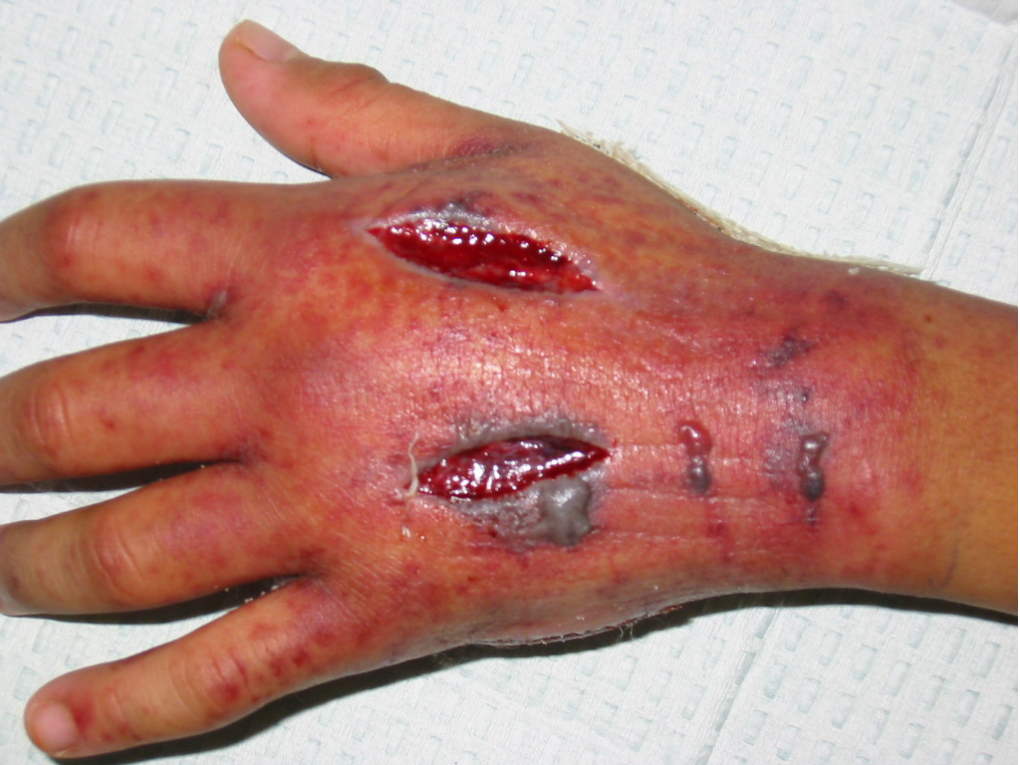
Decompression and fasciotomy of the dorsal interosseous compartments.

**Figure 2:**
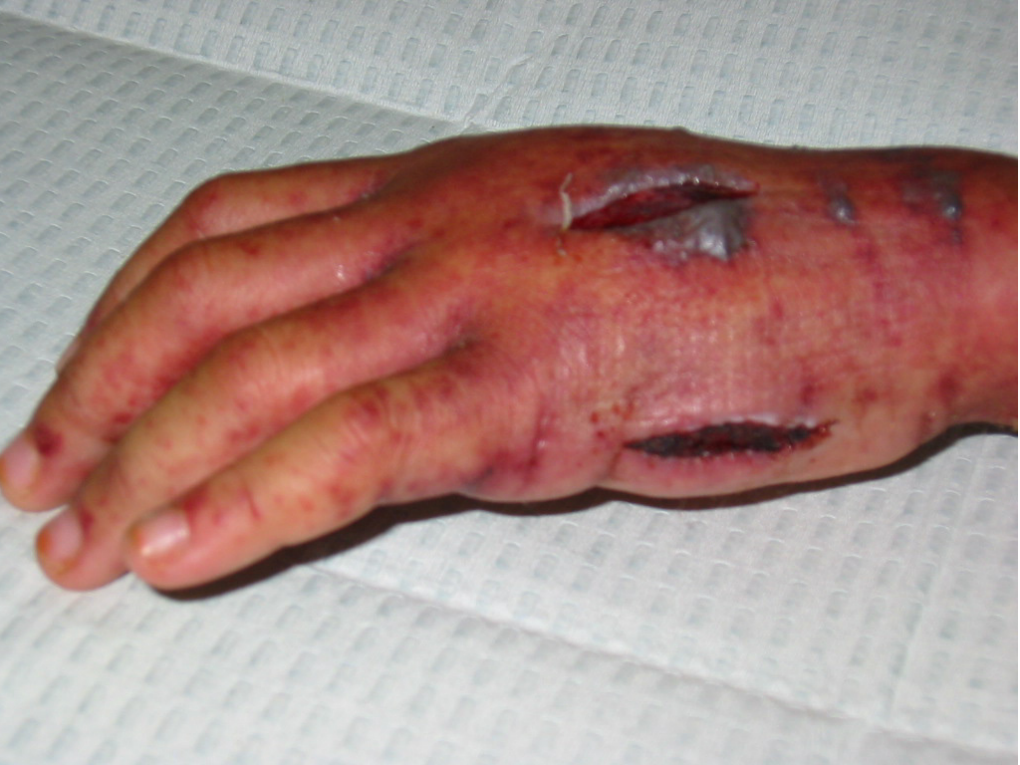
Decompression and fasciotomy of the thenar and hypothenar compartments.

In the late afternoon, the whole left hand was swollen intensely, with extension of the purpura from the hand, up the wrist into the forearm. The metacarpophalangeal joints of all the left fingers were extended with flexion of all the distal interphalangeal joints. Capillary refill was normal. Emergency fasciotomy decompression of the dorsal [Fig 1], thenar and hypothenar compartments [Fig 2] of the left hand were performed. Serous fluid accumulation was noted in all compartments with marked tissue oedema. Additional hematoma was noted in the hypothenar compartment. The rashes and swelling subsided quite quickly and he was discharged uneventfully on the fifth post-operative day.

**Figure 3:**
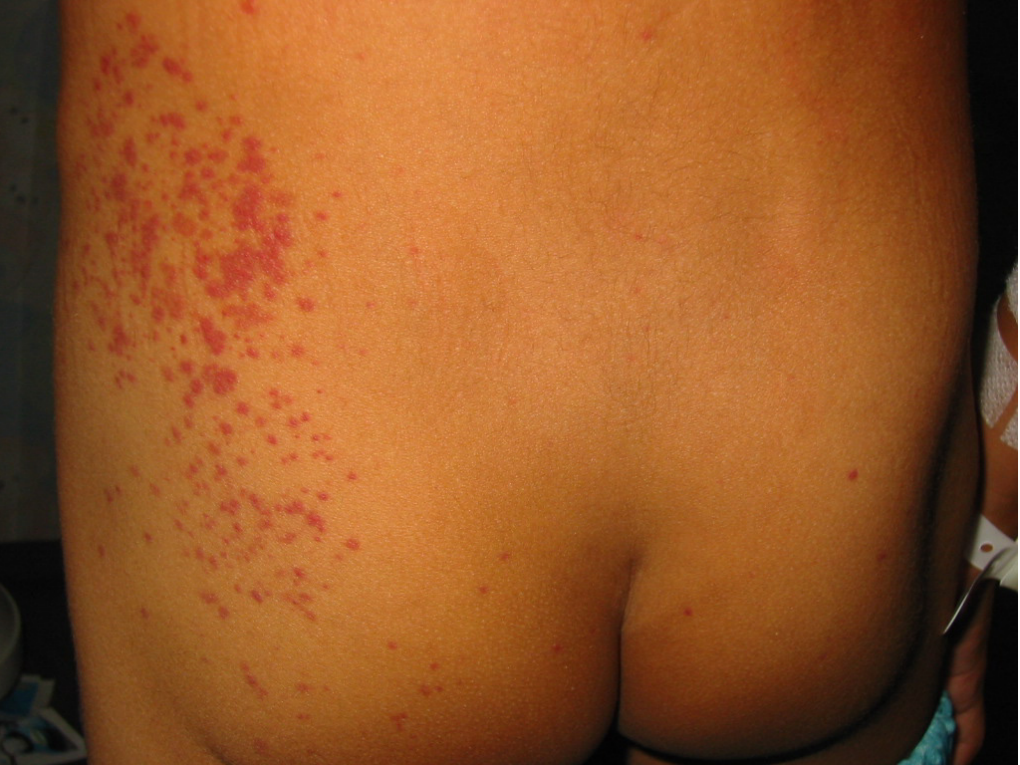
Non-blanching purpura of the left flank and buttock.

## Discussion

This is the first case of acute compartment syndrome of the hand in HSP to be reported in the literature. Complications such as gastrointestinal bleeding [3], intracranial bleeding [4], pulmonary hemorrhages [5], bilateral central artery occlusion [6], orbital hematomas [7], nephritis and penile involvement [8] have been well documented.

While nephritis is the most important determinant of HSP survival outcome, acute compartment syndrome (ACS) of the hand cannot be overlooked since all these patients will need arterial or venous cannulation at some point in their treatments. ACS can lead to ischemia, necrosis and lost of the hand. It is a dire emergency requiring immediate surgical decompression.

Clinical astuteness in the early diagnosis of ACS is extremely important and cannot be overemphasized.

In this case, the severity of purpura correlated well with the severity in hand swelling. There was immediate regression of the purpura following surgical decompression.

It can be hypothesized that in HSP, the initial inflammation and thrombosis of the post-capillary venules in the dermis caused increased exudation, red blood cells extravasation and edema fluid accumulation in the surrounding tissues. The subsequent increase in interstitial pressure exacerbated the vasculitic process, worsened the exudation and purpura. This vicious cycle will eventually culminate in acute compartment pressure.

In the later stage, the inflamed arterioles caused ischemia by compromising the circulation to muscles distal to the cannulation site. This further exacerbated the compartment syndrome.

In HSP, the inflammation and damage occurred primarily in small vessels, especially the post-capillary venules. The following are noted to play major roles in the disease process: 1) complement pathway activation, 2) lymphokines and 3) hemodynamic factors [9].

Arterial cannulation breached the endothelium and caused complement system activation. This resulted in chemotaxis of neutrophils and vessel wall injury with exudation of serum, erythrocytes and fibrin.

Lymphokines such as TNF and IL-1 stimulated the endothelium to activate the intrinsic and extrinsic coagulation pathways and reduce its fibrinolytic activity. This resulted vessel thrombosis.

Hemodynamic factors like turbulence, ischemia and increased venous pressure, as well as reduced fibrinolytic activity that occurred in the legs and flanks, may explain why localisation of the lesions and hence purpura to these sites commonly occur. In this case, the pronounced subepidermal edema also resulted in vesicobullous lesion and skin infarction on the dorsum of his hand.

The high incidence of arterial thrombosis in ICU patients with radial artery cannulation, as shown by Martin et al, must be highlighted to all clinicians. His study (1983) found that in 134 ICU patients with radial cannulation for more than 4 days, no thrombosis was observed in 31 patients (24%), a partial thrombosis was found in 73 patients (57%), and a total thrombosis with vessel obstruction was found in 25 patients (19%) [10].

To prevent the problem of ACS in similar patients, coagulation profiles and bleeding times must be normalized prior to cannulation. Hypoxia and hypotension, if present, must be corrected. There should only be a single attempt at cannulation to prevent hematoma formation.

One should avoid the dorsum of the hand or foot, and away from the wrist and ankle joints, so as to minimize flow turbulence. An open cut-down procedure for cannulation under direct vision is recommended if the intended artery for cannulation proved too small to manipulate.

Finally, a high index of suspicion for acute compartment syndrome is required since the diagnosis is still based on clinical findings. All of us should be alerted to similar cases of ACS in patients requiring arteriovenous fistulae creation, abdominal compartment syndrome in patients requiring CPPD or thoracic outlet syndrome in those with central lines.

## Competing interest declaration

The author(s) declare that they have no competing interests.

## Authors' contributions

Dr ES Ng contributed to the content of this article.
